# Phase-Sensitive Surface Plasmon Resonance Sensors: Recent Progress and Future Prospects

**DOI:** 10.3390/s17122819

**Published:** 2017-12-05

**Authors:** Shijie Deng, Peng Wang, Xinglong Yu

**Affiliations:** State Key Laboratory of Precision Measurement Technology and Instruments, Tsinghua University, Beijing 100084, China; dengshijie@mail.tsinghua.edu.cn (S.D.); jyxyxl@mail.tsinghua.edu.cn (X.Y.)

**Keywords:** surface plasmon resonance, phase detection, heterodyne, ellipsometry, interferometry

## Abstract

Surface plasmon resonance (SPR) is an optical sensing technique that is capable of performing real-time, label-free and high-sensitivity monitoring of molecular interactions. SPR biosensors can be divided according to their operating principles into angle-, wavelength-, intensity- and phase-interrogated devices. With their complex optical configurations, phase-interrogated SPR sensors generally provide higher sensitivity and throughput, and have thus recently emerged as prominent biosensing devices. To date, several methods have been developed for SPR phase interrogation, including heterodyne detection, polarimetry, shear interferometry, spatial phase modulation interferometry and temporal phase modulation interferometry. This paper summarizes the fundamentals of phase-sensitive SPR sensing, reviews the available methods for phase interrogation of these sensors, and discusses the future prospects for and trends in the development of this technology.

## 1. Introduction

Surface plasmon resonance (SPR) is a collective charge density oscillation that occurs at a metal-dielectric interface when light passes through a substrate and is reflected by the metal-dielectric interface [1,2]. If the wave-vector component of the incident light that is oriented parallel to the interface matches the propagation constant of the surface plasmon wave, SPR then occurs. In this case, most of the incident energy is coupled into the surface plasmon mode field, which results in shifts in the resonance angle and wavelength, along with changes in the intensity and phase of the reflected light.

Since SPR was first used for biosensing purposes by Liedberg in 1983 [3], research interest in SPR biosensors has increased rapidly [1,4,5] and these sensors have found wide-ranging applications in fields including drug discovery [6], nucleic acid detection [7], food safety [8], and environmental monitoring [9]. Several methods have been used to date to monitor the excitation of SPR, including angle [10,11], wavelength [12,13], intensity [14,15] and phase [16,17] interrogation techniques. As many researchers have demonstrated [18,19,20,21], the phase of the SPR reflected light changes much more abruptly than its intensity. Therefore, phase-interrogated SPR biosensors are the most sensitive excitation monitoring method.

Because the optical configurations of phase-interrogated SPR sensors are more complex than those of the other sensor types, we believe that it is necessary to review SPR phase detection techniques and to compare the performances of these techniques with the other SPR monitoring methods. This paper focuses on phase-interrogated SPR biosensing technology, reviews the fundamentals of SPR sensing and the associated methods of phase interrogation, and discusses developmental advances and emerging trends in the field.

Prior to the main discussion, it is important to clarify the terminology defining sensitivity characteristics of SPR sensors. The sensitivity of SPR biosensors is composed of chemical and physical sensitivity [10,17]. The chemical sensitivity depends on surface chemistry and assay format (“direct”, “sandwich”, “competitive”, “inhibition” etc.), and the physical sensitivity depends on plasmonic transduction modality, optical configuration, and the level of instrumental and environmental noises. In this review, we will be focused on the methods of SPR response interrogation and thus just consider the physical sensitivity. In addition, the term “sensitivity” usually implies the shift of SPR response (angular or spectral position of the SPR dip, intensity, phase) over the variation of the refractive index (RI). But it is difficult to compare the sensitivity for different interrogation schemes. So here we use the term “sensitivity” to characterize the minimal measurable variation of RI. More exactly, it is should be termed as “limit of detection” (LOD). But since the term “sensitivity” is more prevalent for SPR sensors, we term it as “sensitivity” to compare SPR sensors of different interrogation schemes.

## 2. Principles of SPR Sensing

The theoretical basis of SPR is the interaction between incident electromagnetic waves and the free electrons in a metal. At the interface between a semi-infinite metal layer with complex permittivity $\varepsilon_{m}=\varepsilon_{m}^{\prime }+i\varepsilon_{m}^{\prime \prime }$ and a dielectric medium with complex permittivity $\varepsilon_{d}=\varepsilon_{d}^{\prime }+i\varepsilon_{d}^{\prime \prime }$, where $\varepsilon_{m}^{\prime }$ and $\varepsilon_{d}^{\prime }$ have opposite signs and $\varepsilon_{m}^{\prime }<\varepsilon_{d}^{\prime }$, an incident electromagnetic wave can be coupled to the free electron gas and excite the free electrons to oscillate collectively. Because the behavior of the free electrons is similar to that of a plasma, the collective oscillation is called a surface plasma wave (SPW). Based on an analysis of Maxwell’s equations with appropriate boundary conditions, the wave vector for this SPW can be expressed as [22]:

(1) $$k_{sp}=\frac{2\pi }{\lambda_{0}}\sqrt{\frac{\varepsilon_{m}\varepsilon_{d}}{\varepsilon_{m}+\varepsilon_{d}}}$$

where $\lambda_{0}$ is the wavelength of light in a vacuum. The SPR phenomenon occurs when the wave vector of the SPW, i.e., $k_{sp}$, matches the component of the incident light’s wave vector in the direction parallel to the interface.

**Figure 1:**
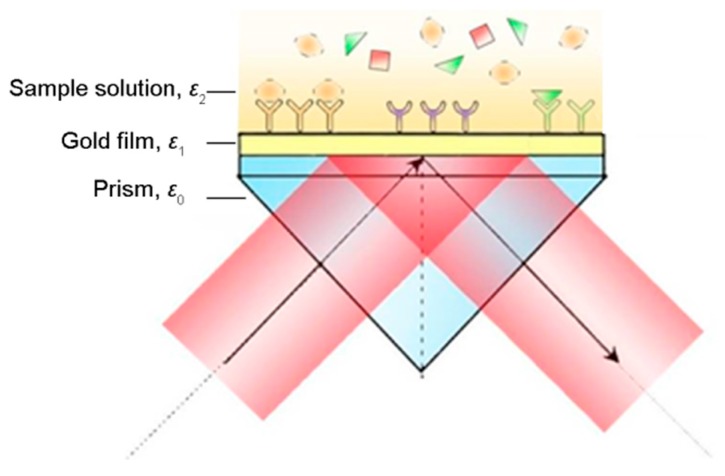
Kretschmann configuration used for surface plasmon resonance (SPR) sensing (Adapted from [24]).

In general, SPR sensing is based on the Kretschmann configuration, which consists of a high refractive index prism, a thin gold film and solution, as shown in Figure 1. Based on a combination of the Fresnel equations and interference theory, the intensity and the phase of the reflected light are determined using the complex reflection coefficient of the multilayer medium structure, and can be expressed as [23]:

(2) $$r_{m}=\frac{r_{0,1}^{m}+r_{1,2}^{m}e^{2idk_{z1}}}{1+r_{0,1}^{m}r_{1,2}^{m}e^{2idk_{z1}}},\;(i=\sqrt{-1},\;m=p,\;s)$$

where the angle of incidence is θ, the thickness of the gold film is *d*, and the dielectric coefficients of the prism, the gold film and the sample solution are $\varepsilon_{0}$, $\varepsilon_{1}$, and $\varepsilon_{2}$, respectively, and

(3a) $$k_{0x}=\frac{\omega }{c}\sqrt{\varepsilon_{0}}\sin\theta$$

(3b) $$k_{zj}=\sqrt{{\left(\frac{\omega }{c}\right)}^{2}\varepsilon_{j}-k_{0x}^{2}},\;\left(j=0,\;1,\;2\right)$$

(3c) $$r_{j,j+1}^{p}=\frac{\frac{\varepsilon_{j+1}}{k_{zj+1}}-\frac{\varepsilon_{j}}{k_{zj}}}{\frac{\varepsilon_{j+1}}{k_{zj+1}}+\frac{\varepsilon_{j}}{k_{zj}}},\;\left(j=0,\;1\right)$$

(3d) $$r_{j,j+1}^{s}=\frac{k_{zj+1}-k_{zj}}{k_{zj+1}+k_{zj}},\;\left(j=0,\;1\right)$$

The intensity and the phase of the reflected light both change when there is a change in the refractive index of the sample solution in the vicinity of the gold film. Intensity monitoring offers the advantage of simple optical configuration requirements, and has been utilized in many commercial devices [25,26]. With a more complex optical configuration, the phase interrogated SPR sensors can obtain a higher sensitivity, since the phase of the reflected light undergoes a more abrupt change than the intensity [18,19,20,27,28,29].

## 3. Optical Configurations for SPR Phase Interrogation

While light intensity measurement is a straightforward process, the high-frequency oscillations (of the order of 10^14^ Hz) of light cannot be observed directly. Complex optical configurations are thus required to retrieve SPR-induced phase changes, with methods including heterodyne detection, ellipsometry and various interferometry techniques [17,30].

### 3.1. Heterodyne Detection

The heterodyne method is commonly used in phase detection [31,32,33]. The fundamental aspect of this method is the generation of two identical laser beams that include two orthogonally polarized components at slightly different frequencies. Combination of these orthogonally polarized components produces an interference signal with a “beat” frequency that is lower than the detector’s response frequency. Extraction of the phase from low-frequency signals then becomes much easier. 

**Figure 2:**
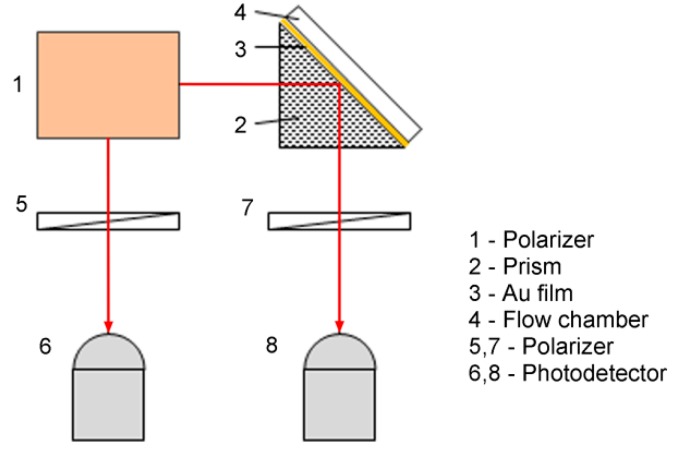
Heterodyne interferometry scheme used for SPR phase interrogation (Adapted from [35]).

The typical optical configuration that is used for SPR sensing is shown in Figure 2. The heterodyne light source emits two identical laser beams; each of these beams includes two orthogonally polarized components at slightly different frequencies. One beam, which is called the reference beam, passes directly through a polarizer to generate an interference signal that has a “beat” frequency that is lower than the detector’s response frequency. The other beam, which is called the measurement beam, passes sequentially through the SPR sensing cell and a polarizer to generate a measurement signal that has the same “beat” frequency but a different initial phase. Comparison of the measurement signal with the interference signal allows the SPR-induced phase change to be retrieved using standard phase detection electronics, such as a phase meter. The key aspect of the use of optical heterodyning for SPR sensing is the generation of a heterodyne light source in which the orthogonally polarized p- and s-components are at slightly different frequencies. Two methods are commonly used to generate the required heterodyne light source, involving use of an acousto-optic modulator (AOM) [34] or a Zeeman laser [35,36,37].

The optical heterodyne-based SPR sensor was initially developed by Nelson et al. in 1996 [38]. In their setup, a linearly polarized beam with frequency $\omega_{1}$ that was emitted by a He-Ne laser was modulated using an acousto-optic modulator (AOM) with a drive frequency of $\omega_{d}=140\;\mathrm{MHz}$ to generate a second diffracted beam with a frequency shift of $\omega_{2}=\omega_{1}+\omega_{d}$. The interference signals were mixed with a local oscillator signal at 140.01 MHz to create easily managed low-frequency signals in the 10 kHz range. Sensitivity as high as 5 × 10^−7^ RIU was expected when their experimental setup was optimized. Shen et al. [35] introduced a frequency-stabilized Zeeman laser as a light source for their optical heterodyne SPR measurement system. This laser source provided two orthogonally polarized components at a frequency difference of 160 kHz without use of optical modulators. Additionally, use of a feedback control strategy produced frequency stability of more than 1 × 10^−7^ RIU. The SPR-induced changes in both the phase and the intensity of the p-polarized light were obtained simultaneously using an electronic phase meter and a voltmeter. The experimental setup was easy to implement and the resulting system was simple to operate. Subsequently, Yu et al. [36] developed an SPR immunosensor that used a similar optical heterodyne configuration. To ease phase retrieval and improve the measurement resolution, the frequency difference in the laser source was reduced to 33.2 kHz with frequency stability of 10^−10^ using a laboratory-developed transverse Zeeman laser. The dynamics of the interactions between ricin and its antibody were researched using this immunosensor.

### 3.2. Ellipsometry

**Figure 3:**
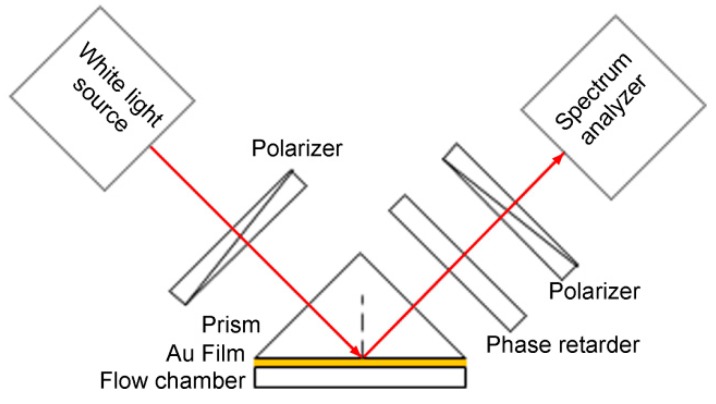
Ellipsometry scheme for SPR phase interrogation (Adapted from [40]).

In the Krestchmann configuration, SPR excitation is associated with dramatic changes in both the phase and the intensity of the p-polarized light, while the s-polarized light remains invariant. This difference between the p- and s-amplitude dependences leads to variation in the reflected light polarization, i.e., the polarization of the reflected light changes when SPR occurs. Ellipsometry is a well-established self-referencing technique that deals with the measurement and interpretation of polarized light that undergoes oblique reflection from a given sample surface [39]. In ellipsometry, as shown in Figure 3, the p- and s-polarized light beams are combined using a polarizer and their relative phase difference is determined through analysis of a series of intensity measurements that have been captured with known angular modulation between them. Ellipsometry provides both an increased dynamic range and high sensitivity because the variations in both the intensity and the phase that are caused by the changes in the resonance conditions of the surface plasmon wave can be analyzed simultaneously. Additionally, the standard calibration procedures in ellipsometric measurements are sufficiently well developed to allow quantitative analyses based on fitting of theoretical models to the experimental results.

The phase polarization properties of light during SPR were actively used before the development of advanced phase-sensitive SPR biosensor designs. As early as 1976, Abeles [39] had derived a theoretical description of the dependence of the phase difference on the wave vector along the surface of the Kretschmann configuration, and proposed the use of ellipsometry to investigate surface or interface reactions. In the 1990s, Herminghaus et al. [41] used the phase polarization properties of light to improve the contrast in SPR microscopy and enable its use in thin film characterization. Kabashin et al. [42] studied the phase and polarization transformations that occurred during SPR and proposed the use of the SPR-related phase jump as a resonance point marker to improve the signal pattern contrast considerably. Piliarik et al. [43,44] used a similar polarization control scheme to observe the spectral SPR features as distinct maxima over a minimum background and thus improve the measurement sensitivity. In 2013, Han and Luo [45] developed a similar ellipsometric SPR system to perform multi-channel measurements and obtained high sensitivity of 1.25 × 10^−6^ RIU. Moreover, Lodewijks et al. [46] investigated localized SPR in randomly distributed gold nanoparticles on top of a continuous gold layer and a dielectric spacer by a spectroscopic ellipsometry, and experimental results showed an increased sensitivity than the intensity measurement.

### 3.3. Interferometry

Interferometry is a widely used optical phase measurement method that provides an important advantage over other phase detection techniques (e.g., ellipsometry, heterodyning) in that it offers the capability to record the phase distribution over the sensor surface. This indicates the possibility that interferometry can be used for mapping of surface reactions and micro-array sensing applications. However, the light that is reflected from the SPR sensing cell cannot interfere with itself. An appropriate optical element was therefore required to create suitable interference conditions. However, the interference state differs when different optical elements are used. At present, the existing interferometry-based SPR sensors can be divided into three types: shear interferometry, spatial phase modulation interferometry and temporal phase modulation interferometry sensors [30,47].

#### 3.3.1. Shear Interferometry

**Figure 4:**
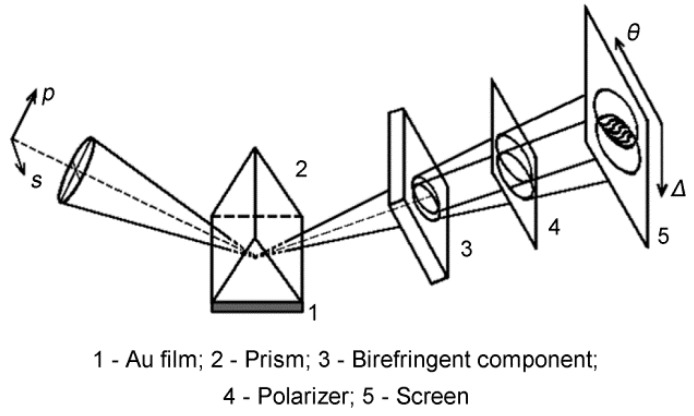
Shear interferometry scheme for SPR phase interrogation (Adapted from [48]).

In 1998, Kochergin et al. [48] first reported visualization of the angular dependence of the phase of the reflected radiation under SPR conditions when they introduced a birefringent component to produce the interference. The SPR shear interferometry scheme is shown in Figure 4. The p- and s-components of the beam that was reflected from the SPR sensing cell were separated in space using a birefringent component. The two components then pass through a polarizer to produce an interference pattern on a screen, where one coordinate represents the phase and the other corresponds to the angle of incidence. The birefringent component ensures that the p- and s-components reach the region in which they overlap on a screen at different angles and the polarizer imposes the same polarization direction on these components. The fringes that were generated showed a kink step near the minimum resonance of the reflected radiation intensity. An inversion in this step was detected during the course of biological binding. The SPR shear interferometry method is compact and is immune to stray noise and drift because it uses the common optical path configuration. A wide dynamic range can also be achieved because of the angular position determination. However, this method is impractical for array detection applications.

#### 3.3.2. Spatial Phase Modulation Interferometry

**Figure 5:**
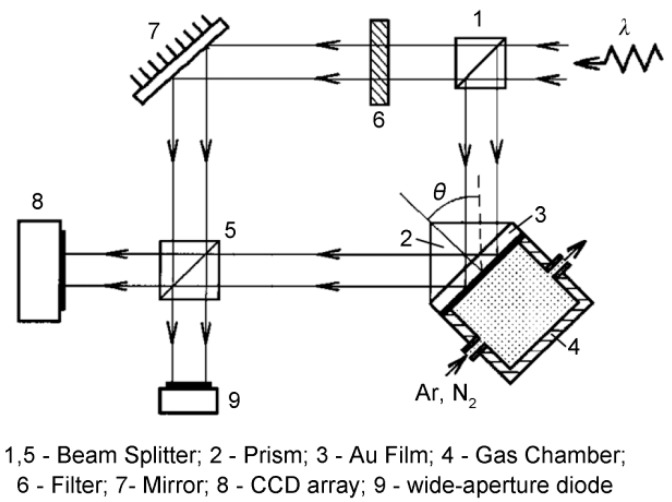
Spatial phase modulation interferometry scheme for SPR phase interrogation (Adapted from [49]).

In 1997, Kabashin and Nikitin [49,50] proposed a device that was based on a Mach–Zehnder interferometer for analysis of the interference pattern to determine the phase shift during SPR. Their method was based on separation of the laser source beam into two components: the measurement beam and the reference beam, as shown in Figure 5. The measurement beam is projected into the SPR sensing cell, where the p-polarized component undergoes a phase shift while the reference p-polarized beam is reflected from the mirror without any phase change. Both beams are then directed at the detector, where the beams interfere to form a specific pattern. The phase shift can then be extracted based on pattern analysis. However, while sensitivity as high as 4 × 10^−8^ RIU was achieved in gas detection applications, the different optical path configurations mean that the interferometry method can easily be disturbed by environmental noise.

Yu and coworkers [51] fabricated a quasi-common-optical-path spatial phase modulation SPR imaging interferometry setup in 2005. In this method, the SPR sensing chip surface is illuminated using a collimated parallel light beam, and the p- and s-polarized components of the reflected light beam are separated using a Wollaston prism. After passing through a polarizer and a set of lenses, the two components of the light interfere at the camera. A local refractive index change of 3 × 10^−5^ RIU was resolved from the phase shift of the interference fringes. Patskovsky et al. [52,53] later proposed a common-optical-path spatial phase modulation scheme. In this scheme, the p- and s-polarized components of the incident beam were spatially modulated when they passed through a birefringent wedge (which acted as a spatial phase retarder). After passing through the wedge, the beam becomes spatially modulated along the wedge axis with periodic changes in the phase relationships between the p- and s-polarized components. The reflected light from the SPR sensing chip is then passed through a polarizer to form interference fringes. Because the measurement beam and the reference beam share a common path, the common optical path system thus has both excellent noise immunity and high detection sensitivity. In later studies, a number of similar SPR sensors were described that used alternative interferometer configurations [30,54,55,56,57,58,59]. 

#### 3.3.3. Temporal Phase Modulation Interferometry

**Figure 6:**
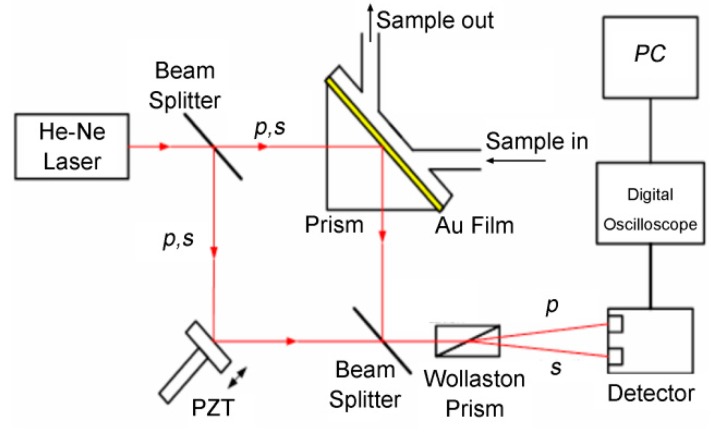
Temporal phase modulation interferometry scheme for SPR phase interrogation (Adapted from [60]).

Spatial phase modulation interferometry requires the formation of a number of interference patterns on the camera to detect the phase of a single sensing spot, which results in limited detection throughput; temporal phase modulation SPR interferometry, however, can theoretically calculate the phase of a single sensing spot from the received light intensity at one charge-coupled device (CCD) pixel. In 2004, Wu et al. [60] developed a scheme using a standard Mach–Zehnder interferometer with a Wollaston prism placed in its output path, as shown in Figure 6. Sequential phase modulation was induced by the periodic movement of the piezoelectric transducer (PZT)-driven mirror. The p-polarization (signal) and the s-polarization (reference) were interrogated simultaneously using the sequential interference patterns. Because the SPR phenomenon only affects the p-polarized light, while the s-polarized light remains unaffected, the SPR phase can be extracted by simply comparing the phase difference between the signal and the reference. Experimental results that were obtained from glycerin-water mixtures showed sensitivity of 5.5 × 10^−8^ RIU, which represents a significant improvement over previously reported results.

Su et al. [61] developed another temporal phase modulation SPR imaging interferometry scheme in 2005. An electro-optic modulator (liquid crystal phase retarder) was arranged in the reflected optical path to induce relative phase variation between the p- and s-polarized components. The reflected beam was thus split into a deflected beam and a straight beam. The former beam was passed through a polarizer that transmits the p-wave only, and this beam was then focused onto a photodiode to detect the SPR angle. Simultaneously, the straight beam, which contained both p- and s-polarized components, was adjusted via the orientation of its optical axis to provide the final contrast. Finally, five sequential interference patterns were acquired using the CCD camera to allow analysis of the phase variation. In addition to its high sensitivity, this setup also provided enhanced temporal stability of 2.5 × 10^−4^ π for 4 h that makes it suitable for long-term monitoring use, regardless of any environmental disturbances, mechanical vibrations, or light source fluctuations. Several similar temporal phase modulation configurations were subsequently developed using a variety of other phase retarders [47,62,63,64,65,66,67].

## 4. Prospects

Despite the complexity of the optical system and the data processing algorithms required, phase-interrogated SPR sensors have been developed rapidly because of their high sensitivity. To date, considerable attention has been paid to detection sensitivity enhancement, expansion of the dynamic range, spatial resolution improvement, and portable and miniaturized sensor design. This section will review these trends.

### 4.1. Sensitivity Enhancement

High sensitivity is always the main goal during instrument development. Following the rapid development of nanotechnology over the past few years, nanomaterials have been incorporated into phase-interrogated SPR sensors for further enhancement of their detection sensitivity [68]. Periodic structures with shapes in metal thin films, including nanodots [69,70], nanorods [71], nanodisks [29], nanoantennas [72], and subwavelength metallic slits [73], have been investigated for their sensitivity enhancement properties. The electric field enhancement produced by coupling of the localized SPR that was excited on the nanomaterial surface with the surface plasmon wave that was excited on the sensing film meant that the sensitivity could be increased by tens to hundreds of times. Additionally, nanoparticles composed of various new materials, including magnetic, carbon-based, latex and liposome particles, have also been widely investigated for this purpose [74,75]. Using the special characteristics of the plasmonic nanostructures, the measurement of the changes in the rotation of light polarization was also investigated for sensitivity enhancement [76,77]. In particular, because graphene has intrinsic plasmons that are tunable and because the combination of graphene with noble metal nanostructures offers a variety of exciting potential applications [78], major efforts have been applied to the development of a combination of graphene with SPR [79,80,81,82].

### 4.2. Dynamic Range Expansion

The expansion of the sensor dynamic range, which has greatly hindered practical application of SPR sensors, is another important current research area [83,84,85]. In 2010, Ng et al. [86] proposed a SPR system that was based on differential spectral interferometry, where the SPR phases of the p- and s-polarized components were acquired simultaneously from the visible-to-near infrared spectrum. This setup effectively combined the most desirable features of the phase-sensitive and spectral SPR techniques. A dynamic range of more than 10^−2^ RIU and a detection limit of 2.2 × 10^−7^ RIU were demonstrated experimentally. Additionally, Ho and coworkers [87,88] developed a novel phase-sensitive SPR setup that was based on temporal modulation of the excitation beam via a photoelastic modulator with subsequent extraction of the phase information at the second and the third harmonics of the modulation frequency. As a result, a dynamic range of more than 0.06 RIU and a detection limit of 2.89 × 10^−7^ RIU were achieved simultaneously. This configuration also offers the added advantage of flexibility for both sensitivity and dynamic range adjustment. In 2015, Mishra et al. [89] proposed a novel prism-based SPR sensor using the Kretschmann configuration that had an extremely large dynamic range, varying from gaseous to that of a high-density liquid, with appreciable sensitivity in the visible region. The prism was made from gallium phosphide (GaP), with its base coated first with gold and then with a thin silicon film. While the SPR configuration used in this research was based on angular interrogation, phase-interrogated SPR configurations may also benefit from use of this novel prism to provide a larger dynamic range.

### 4.3. Spatial Resolution Improvement

Improvement in the spatial resolution of the SPR image is another promising development trend. The lateral resolution in SPR imaging is limited by the propagation length of the SPW that is excited on the surface, which is typically more than a few tens of micrometers [22]. In 1998, Kano et al. proposed a method to excite SPR using light that was focused using an objective lens with a large numerical aperture (NA). While the spatial resolution of the image improved to hundreds of nanometers, the resulting SPR measurement sensitivity was relatively low because they used an intensity-interrogated configuration. Somekh et al. [90] then developed a wide-field high-resolution surface plasmon interference microscopy system. It was composed of a speckle-illuminated Linnik interferometer that behaved in the manner of a wide-field analog of a scanning heterodyne interferometer. This system therefore provided high spatial image resolution and high detection sensitivity simultaneously. Gerber et al. [91] theoretically and experimentally researched surface plasmon polaritons in graphene when reflected and/or scattered by external boundaries (edges), internal boundaries (folds, grain boundaries) and defects using near-field interferometry. The resulting sub-wavelength resolution SPR phase image demonstrated a new degree of freedom for spatial and spectral graphene surface plasmon polariton tuning and modulation for use in optoelectronics applications. Other methods utilizing plasmonic nanostructures were also reported to obtain super-resolution imaging resolutions [92,93,94].

### 4.4. Portable and Miniaturized Design

Portable and miniaturized sensor design is another promising development trend because it offers the prospects of simple measurement systems, low-cost fabrication, and remote sensing capabilities. In 2006, Sepulveda et al. [95] integrated a SPR Mach–Zehnder interferometer into a lab-on-chip system using standard complementary metal-oxide-semiconductor (CMOS) processes. The device has been successfully applied to label-free, real-time detection of the covalent immobilization and hybridization of DNA strands. Similar waveguide interferometer-type SPR devices were reported in [96,97,98]. Another promising approach is based on the use of fiber-optic waveguides [98,99,100,101,102]. For example, Ahn et al. [99] developed a fiber-optic waveguide-coupled SPR sensor, in which the fiber is side-polished and a dielectric waveguide structure sandwiched between two adjacent metal layers is deposited for SPR excitation. This configuration provided both good sensitivity and a high dynamic range. Liu and coworkers [103] developed a fiber-optic SPR biosensor that was based on smartphone platforms. The lightweight optical components and sensing element are connected via optical fibers on a phone case. Therefore, the SPR sensing element can easily be installed in or removed from smartphones. This cost-effective and portable smartphone-based SPR biosensor has many potential applications in medicine, health and environmental monitoring. Because of the complexity of phase-interrogated SPR sensors, most smartphone-based SPR sensors have the wavelength [104,105] or intensity [106,107,108] interrogation configuration. To improve the performance levels of these portable SPR sensors, greater efforts will be required to enable integration of the phase interrogation SPR configuration into a smartphone.

## 5. Conclusions

Phase-sensitive SPR sensors have recently been developed rapidly because of their high sensitivity and high throughput when compared with conventional intensity-, angle- and wavelength-interrogated SPR systems. Many methods have been implemented to realize SPR phase interrogation and each of these methods has both advantages and disadvantages. In future development, improvement of the performance of phase-sensitive SPR sensors will be the main objective, including aspects such as sensitivity enhancement, expansion of the dynamic range, spatial resolution improvement, and portable and miniaturized sensor design. It is anticipated that phase-sensitive SPR sensors will play increasingly important roles in both biological and chemical sensing.

## Abbreviations

The following abbreviations are used in this manuscript:

AOM	acousto-optic modulator
CCD	charge coupled device
CMOS	complementary metal-oxide-semiconductor
PZT	piezoelectric transducer
RIRF	refractive index related factor
RIU	refractive index unit
SPR	surface plasmon resonance
SPW	surface plasma wave
